# Prevalence and Prognosis of Portopulmonary Hypertension in 223 Liver Transplant Recipients

**DOI:** 10.1155/2018/9629570

**Published:** 2018-09-18

**Authors:** Jian Li, Qi Zhuang, Xueming Zhang, Ying Zheng, Zhiqing Qiao, Jianjun Zhang, Xuedong Shen, Jieyan Shen

**Affiliations:** ^1^Department of Cardiology, South Campus, Renji Hospital, School of Medicine, Shanghai Jiao Tong University, Shanghai 201112, China; ^2^Department of Internal Medicine, Shanghai Sixth People's Hospital East Campus, Shanghai University of Medicine & Health Sciences, Shanghai 201306, China; ^3^Department of Cardiology, Renji Hospital, School of Medicine, Shanghai Jiao Tong University, Shanghai 200127, China; ^4^Department of Liver Surgery and Liver Transplantation Center, Renji Hospital, School of Medicine, Shanghai Jiao Tong University, Shanghai 200127, China

## Abstract

**Objective:**

To investigate the prevalence and prognosis of portopulmonary hypertension (PoPH) in liver transplant recipients.

**Methods:**

Patients with advanced liver disease who underwent orthotopic liver transplantation (OLT) were included in this retrospective study from January 2012 to June 2015. According to the 2015 European Society of Cardiology (ESC)/European Respiratory Society (ERS) guidelines for the diagnosis of pulmonary hypertension (PH), patients with tricuspid regurgitation velocity (TRV) >3.4 m/s or 2.9 m/s ≤ TRV ≤ 3.4 m/s coexisting with other echocardiographic PH signs were judged as PH. PH patients with portal hypertension and without other known causes of PH were diagnosed as PoPH.

**Results:**

A total of 223 (170 males and 53 females) middle-aged (50.9 ± 9 years old) liver transplant recipients were included in this study. Fourteen patients (6.3%) were diagnosed with PoPH, and none of the patients were treated with vasodilators before or after OLT. After OLT, patients were followed up for 26 ± 13.5 months. In total, 8 of 14 (57%) PoPH patients died, and the main cause of death was pulmonary infection. Kaplan–Meier survival curves revealed a significant difference in survival between PoPH and non-PoPH patients (*p* < 0.001), and the median survival time after OLT of PoPH was 11.4 months.

**Conclusions:**

The prevalence of PoPH was 6.3% in OLT recipients. The survival of untreated PoPH patients was dismal after OLT.

## 1. Introduction

Pulmonary arterial hypertension (PAH) is a progressively aggravated pulmonary vascular disease that leads to right heart failure and ultimately death. PAH is defined by mean pulmonary artery pressure (mPAP) ≥25 mmHg at rest with normal pulmonary artery wedge pressure (≤15 mmHg) measured by right heart catheterization (RHC) [1]. When PAH occurs in the setting of portal hypertension, the condition is termed “portopulmonary hypertension” (PoPH). Previous studies demonstrated that the prevalence of PoPH was 5-6% in liver transplantation (LT) candidates [2, 3]. In China, PoPH was present in 3.8–10% of cirrhotic patients [4, 5]. LT is a radical cure for advanced liver diseases. In China, hepatitis B virus- (HBV-) related liver diseases represent the most common primary cause for LT [6]. However, the prevalence of PoPH in liver transplant recipients in the Chinese population and the prognosis of these patients are unknown.

The exact pathogenesis of PoPH is unclear. Hyperdynamic state, vasoactive substance imbalance, and other factors may play important roles in the mechanism of PoPH [7]. The initial clinical symptoms of PoPH are not specific, such as dyspnea on exertion and fatigue, and some patients may remain asymptomatic. Transthoracic echocardiography (TTE) is a recommended method to screen for PoPH in patients with advanced liver disease [8]. TTE is a reliable and reproducible tool to estimate cardiac function and hemodynamics [9]. Recently, multiple echocardiographic signs were recommended by the European Society of Cardiology (ESC) and the European Respiratory Society (ERS) for diagnosis of PH [10]. The aims of this retrospective study were to use the diagnostic criteria recommended by ESC/ERS to investigate the prevalence of patients with PoPH in LT recipients and the prognosis of these patients.

## 2. Patients and Methods

### 2.1. Ethics Statement

The study was approved by the Ethics Committee of Renji Hospital, School of Medicine, Shanghai Jiaotong University, Shanghai, China.

### 2.2. Study Population

We retrospectively investigated 223 consecutive patients (170 men and 53 women, with an average age of 50.9 ± 9 years) with advanced liver disease who underwent orthotopic liver transplantation (OLT) in Renji Hospital from January 2012 to June 2015. Patients under 18 years, retransplant patients, and patients with cardiovascular disease (congenital heart disease, valvular heart disease, coronary heart disease, and left heart dysfunction with ejection fraction < 50%), rheumatism, severe lung disease, thromboembolism, kidney disease, active infection, or gastrointestinal hemorrhage (<2 weeks) were excluded.

### 2.3. Clinical and Laboratory Examinations

Clinical data collection included TTE, abdominal ultrasound, and computed tomography (CT) and laboratory examination. As a conventional preoperative examination, TTE was performed by a cardiologist according to the recommendations of the American Society of Echocardiography [11]. Echocardiograms were recorded with patients in the left lateral decubitus position. Standard views were acquired through the parasternal long-axis, parasternal short-axis, and apical four-chamber views. Two-dimensional (2D) echocardiograms, M-mode echocardiograms, and tissue Doppler imaging were performed to measure the size and evaluate heart function.

Tricuspid regurgitation velocity (TRV) and pulmonary regurgitation velocity were measured by continuous-wave Doppler techniques. Right atrial pressure was estimated based on the diameter and the presence of inspiratory collapse of the inferior vena cava [10]. Pulmonary artery diameter (PAD), aortic root diameter (AORD), and left atrial diameter (LAD) were measured from 2D images. Left ventricular end-diastolic diameter (LVDD), left ventricular end-systolic diameter (LVSD), interventricular septal thickness (IVST), and left ventricular posterior wall thickness (LVPWT) were measured using a 2D-targeted M-mode method. Left ventricular ejection fraction (EF) and stroke volume (SV) were calculated with the dedicated automated software EchoPAC, ver. 112 (GE Healthcare, Milwaukee, WI), using the biplane Simpson's method [11]. Cardiac output (CO) = SV ∗ heart rate. Diastolic dysfunction (DDF) was evaluated according to recommendations of the ASE [12].

Other examinations included complete blood cell count, liver function test, renal function examination, and coagulation functions. Severity of liver disease was assessed using Child–Pugh's criteria [13] and Model for End-Stage Liver Disease (MELD) score [14].

### 2.4. Diagnosis of PoPH

Portal hypertension was diagnosed by clinical symptoms (ascites, history of gastrointestinal hemorrhage, abdominal wall varicose veins, and splenomegaly) and expanded portal vein detected by image examination (abdominal ultrasound or CT). According to the recommendation of ESC/ERS, TRV was the main indicator for detecting PH by Doppler TTE [10]. Several additional echocardiographic signs are helpful in judging the probability of PH and are proposed in addition to criteria based on TVR. These echocardiographic PH signs include right ventricle/left ventricle basal diameter ratio >1, flattening of the interventricular septum, early diastolic pulmonary regurgitation velocity >2.2 m/s, pulmonary artery diameter > 25 mm, and inferior cava diameter >21 mm with decreased inspiratory collapse (<50% with a sniff or <20% with quiet inspiration) [10]. Patients with TRV > 3.4 m/s or 2.9 m/s ≤ TRV ≤ 3.4 m/s coexisting with other echocardiographic PH signs were judged to have a high probability of PH. In the present study, PH patients with portal hypertension and without other causes of PH were diagnosed as PoPH. According to the diagnosis, patients were grouped into two groups: PoPH group and non-PoPH group.

### 2.5. Statistical Analyses

All data were analyzed using SPSS 20.0 (SPSS Statistics ver. 20.0, IBM Corporation, Armonk, NY, USA). Variables were expressed as the mean ± SD or median (range) as appropriate, according to their distribution. Categorical variables were displayed as frequencies. Comparisons between two groups were performed using *t*-tests and Mann–Whitney *U*-tests as appropriate. Enumeration and grade data were compared with the chi-square test, Fisher's exact test, and rank sum test, as appropriate. Multivariate binary logistic regression was performed to predict case status. A number of variables that were significantly different (*p* < 0.2) between patients with PoPH and patients without PoPH were included in the logistic regression test. The model was estimated using an “enter” method. The final multivariate model was evaluated by the Hosmer–Lemeshow goodness-of-fit test.

Patients were followed up after OLT for the occurrence of all-cause death until November 2016. Follow-up information was obtained from outpatient follow-up or telephone follow-up. All survival PoPH patients were subject to TTE. Survival curves were generated using the Kaplan–Meier method, and survival between groups was compared by the log-rank test. Figures were generated using GraphPad Prism 5 (GraphPad Software, Inc., La Jolla, CA, USA). Two-tailed *p* < 0.05 was considered significant.

## 3. Results

### 3.1. Demographics

The demographics and clinical features of the included patients are presented in Table 1. In our study, the major cause of liver disease was HBV infection (162 of 223, 72.7%). Among 223 patients, 203 (91%) patients had liver cirrhosis, 129 (57.8%) patients were complicated with portal hypertension, and 105 (47.1%) patients had hepatic carcinoma. Two non-PoPH patients had type 2 diabetes, and 1 PoPH patient had colitis gravis. All patients received tacrolimus/mycophenolate mofetil/Neoral as their immunosuppressive therapy. The type of drug and the dosage were adjusted according to the individual patient's condition during follow-up.

### 3.2. Clinical Features

Fourteen (6.3%) patients were diagnosed with PoPH, and none of the patients were treated with vasodilators before or after OLT (Table 2). Compared with non-PoPH patients, PoPH patients included an increased proportion of females (*p*=0.03). Compared with patients without PoPH, patients with PoPH exhibited increased MELD score (*p*=0.006), Child–Pugh score (*p*=0.001), total bilirubin (*p*=0.005), and aspartate aminotransferase (AST) (*p*=0.02) levels, but lower albumin (*p*=0.01) and Hb (*p* < 0.001) levels (Table 1). No significant differences in age or etiology of liver disease were noted between patients with and without PoPH. The echocardiographic data of patients with and without PoPH are presented in Table 3. No significant differences were noted regarding cardiac function, and chamber size, except PAD.

### 3.3. Risk Factors Analysis

We assessed female, MELD score, Child–Pugh score, albumin, Hb, total bilirubin, and AST in multivariate logistic regression (Table 4). Only reduced Hb levels were independently associated with an increased risk of PoPH. Model fit was adequate as assessed by the Hosmer–Lemeshow goodness-of-fit test (*p*=0.49).

### 3.4. Survival Analysis

**Figure 1:**
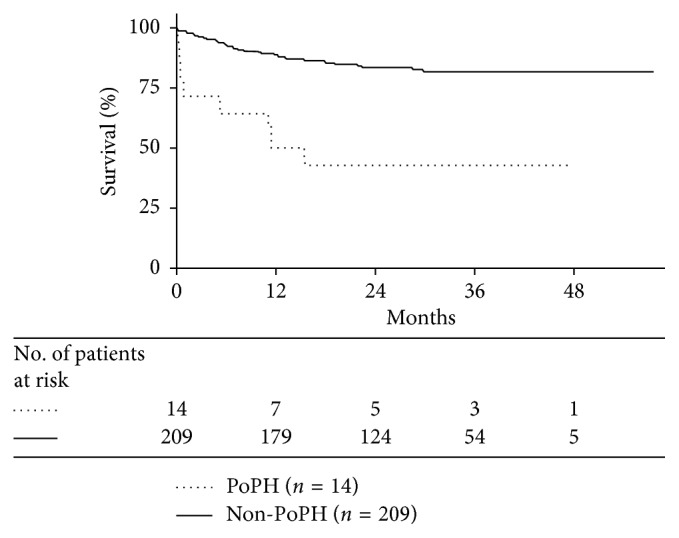
Kaplan–Meier survival curve. There was a lower survival in patients with PoPH compared to patients without PoPH (*p* < 0.001 by the log-rank test). The number of patients at risk at the different times of the follow-up period is reported below the horizontal axis.

Patients who underwent OLT were followed up for 26 ± 13.5 months at the time of termination. During the follow-up period, 8 of 14 (57%) PoPH patients died compared with 38 of 209 (18%) non-PoPH patients. Among PoPH patients, 7 (50%) patients died in the first year after OLT (4 patients died during hospitalization). Of the 8 deceased PoPH patients, 4 patients died of pulmonary infection, 2 patients died of recurrence of hepatocellular carcinoma, 1 patient died of multiple organ failure, and 1 patient experienced an out-of-hospital death of unknown cause. Of the 38 dead non-PoPH patients, 9 patients died of recurrence of hepatocellular carcinoma, 7 patients died of infection, 2 patients died of gastrointestinal hemorrhage, 2 patients died of biliary complications, 1 patient died of kidney failure, and 18 patients experienced out-of-hospital deaths of unknown cause. A significant difference in survival curves was noted between PoPH and non-PoPH patients (*p* < 0.001, HR 16.7, 95% CI 4.27–65.27) (Figure 1). The 6 surviving PoPH patients repeated TTE at the end of the study. Hemodynamics were significantly improved in 5 patients, whereas 1 patient still exhibited increased pulmonary artery pressure (TVR = 3.4 m/s and pulmonary artery systolic pressure (PASP) = 49 mmHg).

## 4. Discussion

In this retrospective study, we first investigated the prevalence of PoPH in liver transplant recipients in a Chinese population and the prognosis of untreated PoPH. A total of 223 OLT recipients were included in our study. In total, 14 (6.3%) patients were diagnosed with PoPH, and a low Hb level was an independent risk factor of PoPH. Our results confirmed the poor prognosis of untreated PoPH patients.

PoPH is an important subgroup of PAH, accounting for approximately 7–10% of PAH cases [15]. TTE is a recommended method of detecting the presence of PoPH in LT candidates [8]. Estimated PASP [16] was the most commonly used variable to detect PH. However, PASP may be underestimated and cannot be used to exclude PH, and overestimation may also occur [10]. Recently, several echocardiographic signs (including right ventricular enlargement, expanded pulmonary artery, abnormal pulmonary flow or regurgitation, and increased right atrial pressure) were recommended in addition to detecting PH based on TRV by ESC/RES [10]. According to this latest guideline for combining TRV with at least one of the echocardiographic signs (Table 2), 14 (6.3%) patients were diagnosed with PoPH in our study. The prevalence was similar to that in previous studies [2].

Regarding clinical presentation, physical examination findings indicate that PoPH symptoms are typically subtle, and greater than half of the PoPH patients are asymptomatic [17]. The clinical features of PoPH have not been adequately described. Among the parameters examined in the present study, patients with PoPH exhibited lower levels of albumin and Hb and increased Child–Pugh and MELD scores compared with patients without PoPH, reflecting more serious liver disease in patients with PoPH. The increased levels of AST and total bilirubin also reflected more serious liver dysfunction in PoPH patients. In our study, most of the included patients have experienced a long course of liver disease and have not been treated effectively in the early stage of disease. The development of PoPH in these patients induces right heart dysfunction, aggravates liver congestion, and exacerbates the original liver disease. Regarding the risk factors of PoPH, our study confirmed findings by Chen et al. [5] demonstrating that low Hb levels were an independent predictor of PoPH. In the present study, compared with non-PoPH patients, PoPH patients included an increased proportion of females. This finding was consistent with previous studies [18]. However, we did not identify differences in the etiology of liver disease between patients with and without PoPH.

In our study, none of the 14 PoPH patients were treated with vasodilators before or after OLT. The survival rate of PoPH patients was reduced. Half of these patients died in the first year, and the median survival time after OLT was 11.4 months. The main cause of death was pulmonary infection. Kaplan–Meier survival analysis revealed that the prognosis of PoPH patients was worse than that of patients without PoPH. The goals of therapy for PoPH are to improve quality of life, improve survival, and facilitate safe and successful LT [19]. LT is the best available therapeutic option for patients with advanced liver disease. LT resolves portal hypertension and is an effective treatment for PoPH. However, without PAH-specific therapy, patients with PoPH still have a poor prognosis after LT [20]. In addition, the failure to resolve PoPH with transplantation suggests irreversible remodeling of the pulmonary artery walls before the initiation of PAH-specific therapy [21]. In our study, one patient exhibited increased PASP after OLT. In recent years, studies have demonstrated that PoPH patients could benefit from vasodilator therapy [20–22], and PoPH patients with vasodilator therapy may have excellent long-term survival after OLT [21]. Vasodilator therapy for PoPH should be considered in all patients with PoPH. Nevertheless, these studies enrolled a limited number of patients with PoPH or were retrospective studies, and further prospective studies are needed to confirm the role of vasodilator therapy in the improvements of hemodynamics and outcomes in patients with PoPH. In the present study, the patient who continued to exhibit increased pulmonary artery pressure was referred to the cardiovascular department for vasodilator therapy.

Our study is a retrospective study. There are some limitations and potentially some bias in our study. RHC is the gold standard for diagnosing PoPH. However, in the present study, no patient underwent RHC before OLT. Nevertheless, the prevalence of PoPH was similar to that in previous studies, and the poor survival in the PoPH group to some extent supported our diagnostic accuracy based on methods of combined multiple echocardiographic signs plus TRV. Although the sample in the present study was large, it was a single-center study, and the number of patients with PoPH was relatively small. These features may explain the lack of difference in the etiology of liver disease between patients with and without PoPH. In addition, the evaluation of prognosis based on this small number of patients may have bias.

In conclusion, the prevalence of PoPH was 6.3% in OLT recipients, and most of these patients had HBV-related cirrhosis. The survival of untreated PoPH patients was dismal after OLT; half of the patients died within a year. It is essential to screen for PoPH in patients with advanced liver disease.

## Figures and Tables

**Table 1:** Demographic and clinical features of all included patients.

Variable	PoPH	Non-PoPH	*p*
Age (years)	53.1 ± 8.7	50.6 ± 8.9	0.31
Gender (male/female), *n*	7/7	163/46	0.03 (Fisher)
Etiology of liver disease, *n* (%)			0.28 (Fisher)
HBV infection	8 (57.1)	153 (73.2)	—
HCV infection	1 (7.1)	9 (4.3)	—
Autoimmune hepatitis	2 (14.3)	17 (8.1)	—
Alcoholic	0 (0)	10 (4.8)	—
Others	3 (21.4)	20 (9.6)	—
With hepatocellular carcinoma, *n* (%)			0.152
Yes	4 (28.6)	101 (48.3)	—
No	10 (71.4)	108 (51.7)	—
Cirrhosis, *n*			0.364
Yes	12	191	—
No	2	18	—
Ascites, *n*			0.249
Yes	7	70	—
No	7	139	—
Portal hypertension, *n*			0.001
Yes	14	115	—
No	0	94	—
Child–Pugh score	9 (6–11)	7 (5–12)	0.001
MELD	20.5 (8–31)	12 (6–40)	0.006
CO (L/min)	6.9 ± 2.8	6.1 ± 1.8	0.27
ALT (IU/L)	35.5 (4–290)	31.5 (6–652)	0.54
AST (IU/L)	78 (13–1098)	46.9 (15.5–602)	0.02
TBIL (mg/dL)	6.9 (1.1–37.9)	2.1 (0.2–54.6)	0.005
Creatinine (mg/dL)	0.7 (0.4–1.3)	0.7 (0.3–80.4)	0.29
ALB (g/L)	30.1 ± 4.5	34.4 ± 6	0.01
Hb (g/L)	85.1 ± 17.9	109.8 ± 25.5	<0.001

HBV, hepatitis B virus; HCV, hepatitis C virus; MELD, Model for End-Stage Liver Disease; ALT, alanine aminotransferase; AST, aspartate aminotransferase; TBIL, total bilirubin; ALB, albumin; Hb, hemoglobin.

**Table 2:** Echocardiography results of the fourteen patients with PoPH.

Case	TRV (m/s)	PAH echocardiographic signs			
		RV/LV basal diameter > 1 or flattening of the interventricular septum	PA diameter > 25 mm	Pulmonary regurgitation velocity > 2.2 m/s	RA pressure ≥ 15 mmHg
1	3.4	−	+	+	−
2	2.9	−	+	+	−
3	3.1	+	+	+	−
4	3.0	+	+	+	−
5	2.9	−	+	+	−
6	3.0	+	+	+	−
7	3.2	+	−	+	−
8	3.1	−	−	+	+
9	3.5	−	−	+	−
10	3.0	−	−	+	+
11	3.1	+	−	+	−
12	2.9	−	+	+	−
13	3.0	−	+	+	+
14	3.1	−	+	+	+

TRV, tricuspid regurgitation velocity; RV, right ventricular; LV, left ventricular; PA, pulmonary artery; RA, right atrium.

**Table 3:** Comparison of echocardiographic data between patients with and without PoPH.

Variable	PoPH	Non-PoPH	*p*
EF (%)	69.3 ± 6.6	67.0 ± 7.4	0.263
CO (L/min)	6.4 ± 2.1	5.8 ± 1.8	0.214
PAD (mm)	27.2 ± 5.5	22.8 ± 3.2	<0.001
AORD (mm)	31.4 ± 3.6	31.2 ± 3.3	0.771
LAD (mm)	39.0 ± 3.8	38.3 ± 5.8	0.503
IVST (mm)	8.0 ± 1.2	8.3 ± 1.3	0.404
LVDD (mm)	51.6 ± 8.6	49.5 ± 5.7	0.198
LVSD (mm)	31.9 ± 6.7	30.9 ± 4.2	0.585
LVPWT (mm)	7.9 ± 0.7	8.2 ± 1.2	0.305
DDF, *n* (%)			0.794
Yes	5 (35.7)	82 (39.2)	—
No	9 (64.3)	127 (60.8)	—

EF, ejection fraction; CO, cardiac output; PAD, pulmonary artery diameter; AORD, aortic root diameter; LAD, left atrial diameter; IVST, interventricular septal thickness; LVDD, left ventricular end-diastolic diameter; LVSD, left ventricular end-systolic diameter; LVPWT, left ventricular posterior wall thickness; DDF, diastolic dysfunction.

**Table 4:** Results of multivariate logistic regression analysis.

Variable	*p*	OR	95% CI
Female	0.06	0.31	0.9–1.069
MELD score	0.66	1.04	0.889–1.205
Child–Pugh score	0.41	1.32	0.681–2.553
ALB	0.63	0.97	0.837–1.114
Hb	0.005	0.96	0.94–0.989
TBIL	0.76	0.99	0.919–1.064
AST	0.47	1.002	0.996–1.008

OR, odds ratio; CI, confidence interval; MELD, Model for End-Stage Liver Disease; ALB, albumin; Hb, hemoglobin; TBIL, total bilirubin; AST, aspartate aminotransferase.
